# Apolipoprotein C3 Promotes Angiogenesis in an Inflammatory Mouse Model of Peripheral Artery Disease

**DOI:** 10.1096/fj.202502155R

**Published:** 2025-09-22

**Authors:** Jordyn M. Thomas, Panashe Bamhare, Jocelyne Mulangala, Christina A. Bursill, Stephen J. Nicholls, Belinda A. Di Bartolo, Kristen J. Bubb

**Affiliations:** ^1^ Department of Physiology, Faculty of Medicine, Nursing and Health Sciences Biomedicine Discovery Institute Clayton Victoria Australia; ^2^ Victorian Heart Institute, Monash University Clayton Victoria Australia; ^3^ Baker Heart and Diabetes Institute Melbourne Victoria Australia; ^4^ Vascular Research Centre, Lifelong Health Theme South Australian Health and Medical Research Institute Adelaide South Australia Australia; ^5^ Western Sydney Area Health Service Westmead Hospital Sydney Australia; ^6^ Adelaide Medical School University of Adelaide Adelaide South Australia Australia; ^7^ Victorian Heart Hospital Monash Health Clayton Victoria Australia

**Keywords:** apolipoprotein C‐III, endothelial cells, hypoxia‐inducible factor 1 alpha subunit (HIF‐1α), inflammation, macrophages, pathological angiogenesis, peripheral arterial disease, physiologic neovascularization, triglycerides, vascular endothelial growth factor A (VEGFA)

## Abstract

Apolipoprotein C3 (ApoC3) regulates triglyceride metabolism and is associated with accelerated atherogenesis and adverse cardiovascular outcomes. However, its role in peripheral artery disease (PAD) remains unclear. We investigated whether *Apoc3* deficiency impacts key features of PAD. Vascularization was assessed using an inflammatory periarterial cuff model (21 days) and a hind limb ischemia model (14 days) in male and female *Apoc3* ^+/+^ and *Apoc3* ^−/−^ mice. Neovascularization was also assessed in mice following extracellular matrix (ECM) plug implantation. Isolated human umbilical vein endothelial cells (HUVECs) were co‐cultured with ApoC3‐stimulated THP‐1 monocytes, and tubule formation was assessed. *Apoc3*‐deficient mice demonstrated less neovessel formation around the cuffed femoral artery, with endothelial cell (CD31+) staining reduced by approximately 40% compared to *Apoc3* ^+/+^ mice. Twenty‐four hours after cuff placement, *Apoc3* ^+/+^ vessels exhibited increased expression of angiogenic (*Hif1a* and *Vegf1*) and pro‐inflammatory (*Cd68*) markers, while *Apoc3*‐deficient vessels did not. Confirming a role for inflammation in ApoC3‐induced angiogenesis, tubulogenesis of HUVECs increased only in the presence of ApoC3 and THP‐1 monocytes. *Apoc3* deficiency, however, did not affect ischemia‐driven angiogenesis, as there were no differences in revascularization compared to *Apoc3* ^+/+^ mice, as assessed by the perfusion index (laser Doppler), fibrosis (Picrosirius red staining), or the mRNA expression of apoptotic (*Bax*), angiogenic (*Hif1a* and *Vegf1*), and inflammatory (*Ccl2*, *Il6*, and *Vcam1*) markers in the ischemic hind limb. Neovascularization following ECM plug implantation was also unaffected by *Apoc3* deficiency. In conclusion, ApoC3 contributes to pathological, inflammation‐driven angiogenesis, highlighting its potential as a therapeutic target for pathological angiogenesis without inhibiting physiological ischemia‐driven angiogenesis.

## Graphical abstract

Apolipoprotein C3 (ApoC3) promotes inflammatory, but not physiological, angiogenesis. ApoC3 increased tube formation and VCAM1 expression in vitro and enhanced VEGF expression in a peri‐arterial cuff (cuff) model of inflammatory angiogenesis. However, ApoC3 did not alter VEGF‐HIF‐1α–mediated angiogenesis in the hind limb ischaemia (HLI) model of ischaemic angiogenesis or extracellular matrix (ECM) models. ApoC3 deficiency (*Apoc3* ^−^/^−^) reduced inflammatory angiogenesis, suggesting a selective role for ApoC3 in inflammation‐driven neovascularisation relevant to peripheral artery disease pathophysiology.

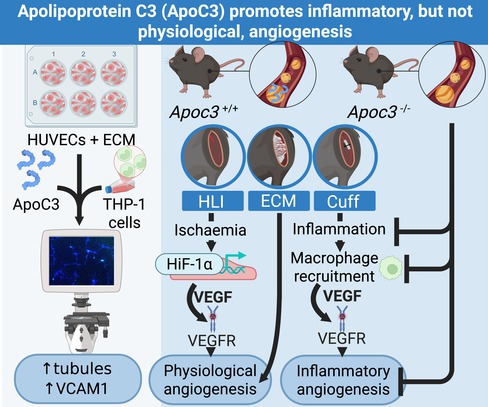

## 1 Introduction

Peripheral artery disease (PAD) increasingly contributes to morbidity and mortality among the global population, affecting > 200 million people worldwide [1]. PAD commonly affects adults over 70 years old, occurring mostly in the lower limbs, and causes significant pain and disability due to poor blood flow. Standard modifiable risk factors for cardiovascular disease (CVD) contribute to PAD, including smoking, high blood pressure, inactive lifestyles, and diabetes mellitus. Atherosclerotic lesions in the femoral arteries are key to the disease progression and severity. Evidence‐based treatments aimed at slowing PAD include smoking cessation, antiplatelet and antihypertensive therapies [2], exercise therapy [3], and lipid‐lowering strategies. When the disease severity reaches critical limb‐threatening ischemia, there is often a need for limb‐salvaging strategies (i.e., endovascular therapies and surgical amputation). There is an unmet need to improve treatments for PAD to avoid limb loss and chronic pain.

Angiogenesis, which involves the growth of new vessels from existing vessels, is crucial for tissue revascularisation in response to ischemia caused by atherosclerotic occlusions [4]. Therapies that promote neovascularisation around peripheral arterial lesions are a rational approach to improving lower limb reperfusion and preventing amputations. Much research into PAD treatment has focused on harnessing endogenous pro‐angiogenesis pathways to improve perfusion in the ischemic limb. Not only can new blood vessel development improve blood flow to reduce pain from obstructive ischemia, the contribution to muscle regeneration can also aid ambulation [5]. Movement and exercise are key to overcoming symptoms of PAD [3], and therefore stimulation of angiogenesis might help improve exercise capacity as an additional benefit [5]. Vascular endothelial growth factor (VEGF) is a key regulator of physiological angiogenesis, promoting endothelial cell proliferation, migration, and tube formation [6, 7, 8, 9]. Despite its central role in angiogenesis, therapeutic approaches targeting the VEGF pathway have yielded limited success in clinical trials for PAD [10, 11]. Inflammatory stimuli can trigger nuclear factor kappa‐light‐chain‐enhancer of activated B cells (NF‐κB)‐dependent VEGF expression [12], potentially driving pathological angiogenesis. This has been associated with both atherosclerotic plaque formation and cancer. Notably, neovascularisation within plaques is increasingly recognized as a contributing factor to plaque instability. Therefore, there is an intriguing balance between physiological and pathophysiological angiogenesis that must be achieved, and this could prove important in PAD where chronic ischemia and inflammation are factors.

Apolipoprotein C3 (ApoC3) was initially recognized for its role in promoting hypertriglyceridemia. Elevated levels of circulating ApoC3 are associated with dyslipidemia, insulin resistance, and increased cardiovascular risk [13, 14, 15, 16]. ApoC3 partly inhibits triglyceride catabolism through lipoprotein lipase (LPL)–dependent and LPL‐independent pathways [17]. The association between elevated plasma triglyceride levels and CVD risk is established [18]. Hypertriglyceridemia increases the risk of developing both type 2 diabetes and PAD/chronic wounds. This raises the possibility that a reduction in ApoC3 will slow atherosclerosis progression and prevent vascular ischemia.

Vascular inflammation is critically involved in the onset and progression of PAD, contributing to endothelial dysfunction and initiation of atherosclerosis [19]. There is accumulating evidence that ApoC3 also contributes to inflammation [20, 21]. ApoC3 activates the NF‐κB pathway and induces monocyte/macrophage activation and adhesion to endothelial cells [22]. This suggests ApoC3 has triglyceride‐independent effects on the vasculature, and lowering ApoC3 might have multiple advantages in diseases of metabolic disturbance and vascular ischemia. To date, the role of ApoC3 in angiogenesis has not been studied. This study aimed to determine whether ApoC3 is a key regulator of angiogenesis by independently assessing the effect of *ApoC3* deficiency on angiogenesis induced by either chronic vascular inflammation or by chronic ischemia. To achieve this, we used two mouse models that recapitulate different aspects of endogenous angiogenesis in the femoral vascular bed: (i) a periarterial femoral cuff model, which has previously been shown to induce neo‐vascularization, expression of chemoattractant molecules, and macrophage infiltration around the cuffed vessel after 21 days [23, 24]; or (ii) a hindlimb ischemia model, which is a classic model of collateral vessel development by angiogenesis and arteriogenesis [25] and demonstrates gradual reperfusion over the 14‐day period in mice on a C57/B6 background [26].

## 2 Materials and Methods

### 2.1 Animal Studies

A total of 67 male or female wild type (*Apoc3* ^+/+^) or *Apoc3* ^−/−^ mice (B6.129‐Apoc3tm1Unc/J; RRID:IMS_JAX:002057), backcrossed ten times onto a C57Bl/6 background, were used for this study. Mice were aged 8–15 weeks and weighed 18–37 g (Table 1). Mice were obtained from either the Monash Animal Research Platform (MARP; Monash University, Australia) or the South Australian Health and Medical Research Institute (SAHMRI). Prior to surgery, mice were housed with littermates in groups of 3–4 animals, on a 12 h light–dark cycle, and provided with ad libitum access to normal chow and drinking water. Environmental enrichment included nesting materials such as tissues and shelters. All procedures were conducted according to the Australian Code for the Care and Use of Animals for Scientific Purposes (8th edition) and approved by the South Australian Health and Medical Research Institute (SAM186) and Monash University (Project number: MARP/22704 and MARP/36928) Animal Ethics Committees.

**TABLE 1:** Mice used for in vivo studies.

	Mouse strain	Origin	No. of mice	Age at treatment (weeks)	Weight at treatment (g)	Deaths
Femoral cuff model	*Apoc3* ^−/−^	SAHMRI	13	8–12	24.10 ± 0.70	
	*Apoc3* ^+/+^		13	8–12	24.68 ± 0.72	
Hind limb ischemia model	*Apoc3* ^−/−^	MARP	10	11–15	30.14 ± 1.633	2^a^
	*Apoc3* ^+/+^		7	13–15	30.49 ± 0.7304	
Matrigel plug model	*Apoc3* ^−/−^		13	8–13	23.26 ± 1.236	
	*Apoc3* ^+/+^		11	8–13	22.75 ± 0.9654	

^a^ Post‐surgical complications.

### 2.2 Murine Periarterial Cuff Model

The peri‐arterial cuff model of inflammatory‐driven angiogenesis was used for this study wherein the femoral artery was isolated from the neurovascular bundle and a 2.0 mm non‐occlusive polyethylene cuff made of PE‐50 tubing (427 410; BD Bioscience, MA, USA) was placed around the left femoral artery of each mouse. The right femoral artery was used as an internal control. All surgeries were performed under anesthesia induced by inhalation of isoflurane (3%). Anesthesia was maintained by 2% isoflurane and was regularly monitored by checking hind‐paw withdrawal, blink reflexes, and respiratory rate. Prior to surgery, mice received local anesthetic (bupivacaine; 2.5 mg/kg, s.c.) and opioid analgesic (buprenorphine; 0.1 mg/kg, s.c.) and were treated with an additional analgesic (carprofen; 5 mg/kg, s.c.) following surgery.

### 2.3 Murine Hindlimb Ischemia Model

The hindlimb ischemia model of hypoxia/ischemia‐mediated angiogenesis was also used for this study wherein the femoral vascular bed was isolated from the neurovascular bundle, and the proximal and distal ends of the left femoral vein and artery were ligated with 6‐0 silk suture and completely excised [26]. The right hindlimb was used as an internal control. All surgeries were performed under anesthesia induced by inhalation of isoflurane (3%). Anesthesia was maintained by 2% isoflurane and regularly monitored by checking hind‐paw withdrawal, blink reflexes, and respiratory rate. Prior to surgery, mice received local anesthetic (bupivacaine; 2.5 mg/kg, s.c.) and opioid analgesic (buprenorphine; 0.1 mg/kg, s.c.) and were treated with additional analgesic (carprofen; 5 mg/kg, s.c.) following surgery. Blood reperfusion of the hindlimb was determined by MoorO_2_FLO laser speckle contrast (Moor Instruments, Axminster, Devon, UK) under anesthesia at baseline, post‐surgery, and on days 3, 7, 10, and 14 following surgery.

### 2.4 Measurement of mRNA Expression Levels

#### 2.4.1 Peri‐Arterial Cuff Model

An additional cohort of mice was used to assess changes in mRNA expression of key angiogenesis and inflammation genes in cuffed and non‐cuffed arteries. Tissue was collected after 24 h to determine the early response to the cuff. Femoral arteries were homogenized, and total RNA was extracted using TRI reagent (Sigma‐Aldrich, St. Louis, MO, USA) and quantified spectrophotometrically. For all RNA samples, the absorbance ratio (*A*_260_/*A*_280_) was within the 1.8–2.0 range and normalized to 100 ± 3 ng/mL. Total RNA (300 ng/μL) was reverse transcribed to generate complementary DNA (cDNA) using the iScript cDNA synthesis kit (Bio‐Rad, Hercules, CA, USA). Quantitative real‐time PCR was performed on cDNA in triplicates using SSoAdvanced Universal SYBR Green Supermix in a CFX Connect Real‐Time System (Bio‐Rad, Hercules, CA, USA; RRID:SCR_026760).

#### 2.4.2 Hindlimb Ischemia Model

Snap‐frozen gastrocnemius muscle from the 14‐day timepoint was pulverized, and RNA was extracted using an RNeasy Mini Kit (Qiagen, Hilden, North Rhine‐Westphalia, Germany). RNA was reverse transcribed (QuantiTect Reverse Transcription Kit; Qiagen, Hilden, North Rhine‐Westphalia, Germany), and the resulting cDNA was then used as a template in real‐time PCR using QuantiTect SYBR Green Master Mix (Qiagen, Hilden, North Rhine‐Westphalia, Germany) in a QuantStudio 7 Real‐Time PCR system (ThermoFisher Scientific, USA; RRID:SCR_020245).

Genes of interest included angiogenic markers: *Bax*, hypoxia‐inducible factor 1*a* (*Hif1a*) and vascular endothelial growth factor 1 (*Vegf1*) and inflammatory markers: C‐C motif ligand 2 (*Ccl2*), interleukin‐6 (*Il6*), *Vcam1*, *Rela*, and *Cd68*, and *36B4* or *βactin* were used as housekeeping genes. The comparative Ct method was used to calculate fold changes in mRNA expression relative to a reference sample [27].

### 2.5 Plasma Analyses

All plasma was collected at the conclusion of the 21 or 14 day protocols. LPL activity was determined using the Lipoprotein Lipase Activity Assay kit (ab204721; Abcam, Cambridge, MA, USA). Prior to euthanasia, mice were injected with 0.2 U heparin/g of body weight via tail vein injection. Plasma was isolated by centrifugation at 3000 rpm for 15 min of whole blood collected into heparinised tubes. In a 96 well plate, 10 μL of mouse plasma (adjusted to 50 μL with ddH_2_O) was added to 50 μL of diluted substrate, and standard was added to 50 μL of reaction mix. Plates were incubated at 37°C for 10 min, protected from light, and read at Ex/Em = 500–550/478 using the GloMax Microplate Reader (Promega, Madison, WI, USA; RRID:SCR_026323), every 10 min for at least 1 h at 37°C. LPL activity for unknown samples was interpolated from the standard curve.

Total cholesterol concentration was determined using the Cholesterol E kit (439‐17501; Wako Diagnostics, Mountain View, CA, USA). In a 96‐well plate, 2 μL of mouse plasma or standard was added to 250 μL color reagent and incubated at 37°C for 5 min. Plates were quantified at 600 nm using the GloMax Microplate Reader. Total cholesterol concentrations for unknown samples were interpolated from a standard curve.

Triglyceride levels were determined using the Triglyceride E kit (432‐40201; Wako Diagnostics, Mountain View, CA, USA). In a 96‐well plate, 2 μL of mouse plasma or standard was added to 250 μL color reagent and incubated at 37°C for 5 min. Plates were quantified at 600 nm using the GloMax Microplate Reader. Total triglycerides for unknown samples were interpolated from the standard curve.

### 2.6 Immunohistochemistry

At the end of the 21‐day femoral cuff protocol, mice were euthanized by cardiac exsanguination and perfused with saline (10 mL) via the left ventricle. The femoral arteries of each leg were excised for histological analyses, fixed in 4% (v/v) paraformaldehyde, paraffin embedded, and cut into 5 μm sections. Following sodium citrate antigen retrieval (pH 6; AJAX Finechem, Taren Point, NSW, Australia), artery sections were deparaffinized, rehydrated, and probed for neo vessel (CD31^+^) or arteriole (smooth muscle actin) formation. Sections were blocked in hydrogen peroxide solution (0.3% H_2_O_2_ in methanol) and goat serum (10% [v/v] goat serum in PBS) and incubated overnight at 4°C with rabbit anti‐CD31 (1:25, ab28364; Abcam, Cambridge, MA, USA; RRID:AB_726362) or mouse anti‐smooth muscle α‐actin conjugated to alkaline phosphatase (1:100, A5691; Sigma‐Aldrich, St. Louis, MO, USA; RRID:AB_476746). Primary antibodies were detected using horseradish peroxidase (HRP) secondary antibody (1:200, ab6721; Abcam, Cambridge, MA, USA; RRID:AB_955447) and 3,3′‐diaminobenzidine (DAB) substrate (Vector Laboratories, Burlingame, CA, USA) or Vector Red alkaline phosphatase substrate (SK‐5100; Vector Laboratories, Burlingame, CA, USA) as appropriate. Cell nuclei were counterstained with hematoxylin, and images were captured using a Carl Zeiss Axio microscope and AxioCamER camera using the ZEN lite software (Oberkochen, Baden‐Württemberg, Germany). One high‐magnification field of view was taken per section for analysis.

### 2.7 Histopathology Staining

At the end of the 14‐day hind limb ischemia protocol, mice were euthanized by cardiac exsanguination, and the gastrocnemius muscles of both the ischemic and non‐ischemic hindlimbs were isolated for histological and mRNA expression analyses. Formalin (10%) fixed, paraffin‐embedded gastrocnemius sections (4 μm) were stained with either Picrosirius red or hematoxylin and eosin by the Monash Histology Platform. Slides were imaged using an Aperio Slide Scanning Unit (Leica Biosystems, Wetzlar, Hesse, Germany), and images (20× magnification) were captured using Aperio ImageScope Software (Version 12.4.6.5003; Leica Biosystems, Wetzlar, Hesse, Germany; RRID:SCR_020993). Picrosirius red staining was quantified to determine percentage collagen content and muscle fiber number, and centralized nuclei were determined from hematoxylin and eosin staining by ImageJ.

### 2.8 Extracellular matrix (ECM) Plug Neovascularisation

Mice were injected subcutaneously with 9 mg/mL reduced‐growth factor extracellular matrix (ECM; Cultrex; R&D Systems, Minneapolis, MN, USA) containing 400 ng/mL FGF‐2 (Sigma‐Aldrich, St. Louis, MO, USA) and 50 U heparin, which formed a solid plug at body temperature. After 14 days, plugs were extracted and homogenized in 0.5 mL of cell lysis buffer and centrifuged at 6000 *g* at 4°C for 30 min. Hemoglobin was detected in the supernatant using a colorimetric assay (Sigma‐Aldrich, St. Louis, MO, USA) at 400 nm wavelength [28].

### 2.9 Endothelial 3‐Dimensional Tube Formation Assay

Human umbilical vein endothelial cells (HUVEC; 1 × 10^4^ cells; Lonza, Basel, Basel‐Stadt, Switzerland) were seeded on a 96‐well plate containing reduced‐growth factor ECM (Cultrex; R & D Systems, Minneapolis, MN, USA) for 4 h at 37°C and 5% CO_2_. Tubule formation in HUVECs was then measured after co‐culture with human monocytic THP‐1 cells (2.5 × 10^4^ cells; ATCC, Manassas, VA, USA; RRID:CVCL_A4CA), which had been pre‐treated for 30 min with ApoC3 (50 μg/mL; Sigma‐Aldrich, St. Louis, MO, USA), LPS (50 μg/mL), or ATP (5 mM) in reduced serum EGM2 (Lonza, Switzerland) for 4 h. Tubule formation was determined by measuring branch number using ImageJ software (National Institutes of Health, Bethesda, MD, USA; RRID:SCR_002285). Cells were fixed in situ with 4% formalin, permeabilized for staining with 1 × PBS containing 0.25% Triton X‐100 (Sigma‐Aldrich; St. Louis, MO, USA), and blocked with casein. Cells were incubated with rabbit anti‐vascular cell adhesion molecule 1 (VCAM‐1, 1:500, ab134047; Abcam, Cambridge, MA, USA; RRID:AB_2721053) for 1 h at room temperature and detected using Alexa Fluor 488‐conjugated goat secondary antibodies (Invitrogen, Carlsbad, CA, USA; RRID:AB_143165). Cell nuclei were counterstained with 4′,6‐diamidino‐2‐phenylindole (D1306; Invitrogen, Carlsbad, CA, USA). Fluorescent images were captured using an ECLIPSE Ts2‐FL inverted microscope (Nikon, Shinagawa, Tokyo, Japan). The number of VCAM‐1–positive tubules was determined by J.M.T and K.J.B, both blinded to treatment grou, and the final value was calculated as the average of their observations.

### 2.10 Statistics

Unless stated otherwise, results are expressed as mean ± SEM. Comparisons between treatment groups were calculated using either unpaired *t*‐test or one‐ or two‐way ANOVA with Tukey's comparison test post hoc, as appropriate. *p* values < 0.05 were considered statistically significant.

## 3 Results

### 3.1 Global Deletion of ApoC3 Reduces Circulating Triglycerides Independently of LPL Activity

Assessment of plasma lipids showed *Apoc3* ^−/−^ mice to have lower triglyceride levels compared to *Apoc3* ^+/+^ mice (Table 2). These differences in triglyceride levels were observed, despite no difference in LPL activities in the presence or absence of ApoC3 (Table 2). There was no difference in total cholesterol levels between *Apoc3* ^−/−^ and *Apoc3* ^+/+^ mice (Table 2).

**TABLE 2:** Lipid profile and LPL activity of mice.

	Mouse strain	Total cholesterol (mg/mL)	Total triglycerides (mg/mL)	Lipoprotein lipase activity
Femoral cuff model	*Apoc3* ^−/−^	0.75 ± 0.06	0.42 ± 0.02	22.81 ± 3.54
	*Apoc3* ^+/+^	0.83 ± 0.06	0.74 ± 0.05	19.16 ± 1.80
	*p*	0.37	< 0.0001	0.38
Hind limb ischemia model	*Apoc3* ^−/−^	0.58 ± 0.01	0.64 ± 0.08	9.29 ± 0.85
	*Apoc3* ^+/+^	0.62 ± 0.01	1.24 ± 0.21	8.16 ± 0.31
	*p*	0.03	0.01	0.33

*Note:* Statistical comparisons were determined by an unpaired *t*‐test. Data is expressed as mean ± SEM.

### 3.2 ApoC3 Global Deletion Blunts Inflammatory‐Induced Adventitial Neovascularization In Vivo

**FIGURE 1:**
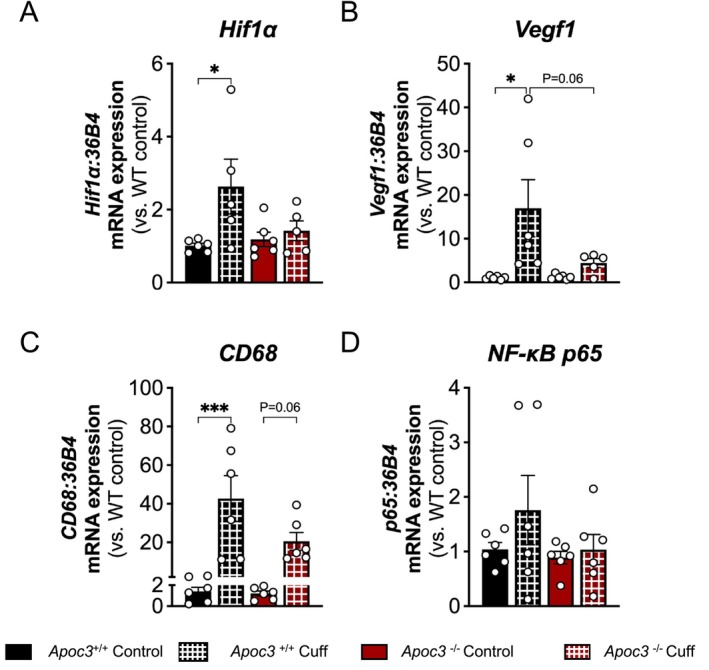
*Apoc3* ^−/−^ mice are protected from peri‐arterial cuff‐induced angiogenic gene expression. mRNA expression of (A) *Hif1a*, (B) *Vegf1*, (C) *Cd68*, and (D) *Rela* in cuffed (cuff) and control femoral arteries from *Apoc3* ^+/+^ and *Apoc3* ^−/−^ mice as measured by real‐time PCR. Values are expressed as mean ± SEM (*n* = 6 per group). ***p ≤ 0.001 and *p ≤ 0.05 for two‐way ANOVA followed by Tukey's post‐tests.

To explore the role of ApoC3 in inflammation‐induced angiogenesis, we employed a femoral cuff model, which involves placing a non‐occlusive cuff around the left femoral artery. One day post‐cuff placement, the mRNA expression of key angiogenic genes *Hif1a* (2.6‐fold, Figure 1A) and *Vegf1* (16.9‐fold, *p* < 0.05, Figure 1B) was augmented in the cuffed femoral arteries of *Apoc3* ^+/+^ mice compared to uncuffed femoral arteries. In contrast, in cuffed arteries of *Apoc3* ^−/−^ mice, the increase in *Hif1a* and *Vegf1* mRNA expression was not significantly different from non‐cuffed (1.4‐fold increase and 4.4‐fold increase, respectively; Figure 1A,B) and trended lower than Apoc3^+/+^ cuffed mice (*p* = 0.06).

The increased mRNA expression of the key inflammatory gene *Cd68* in the cuffed femoral arteries of *Apoc3* ^+/+^ mice followed a similar pattern at one day post‐surgery, with expression increasing ~30‐fold compared to control (*p* ≤ 0.001, Figure 1C). Although *Cd68* expression was also elevated in *Apoc3* ^−/−^ mice (~17‐fold increase in cuffed vs. control arteries), this increase was not statistically significant and was lower when compared to *Apoc3* ^+/+^ cuffed vessels (*p* = 0.06). Expression of *Rela*, the gene that encodes the NF‐κB p65 subunit, tended to increase in the cuffed femoral arteries of *Apoc3* ^+/+^ mice compared to control (Figure 1D); however, cuff placement failed to induce its expression in the femoral arteries of *Apoc3* ^−/−^ mice.

**FIGURE 2:**
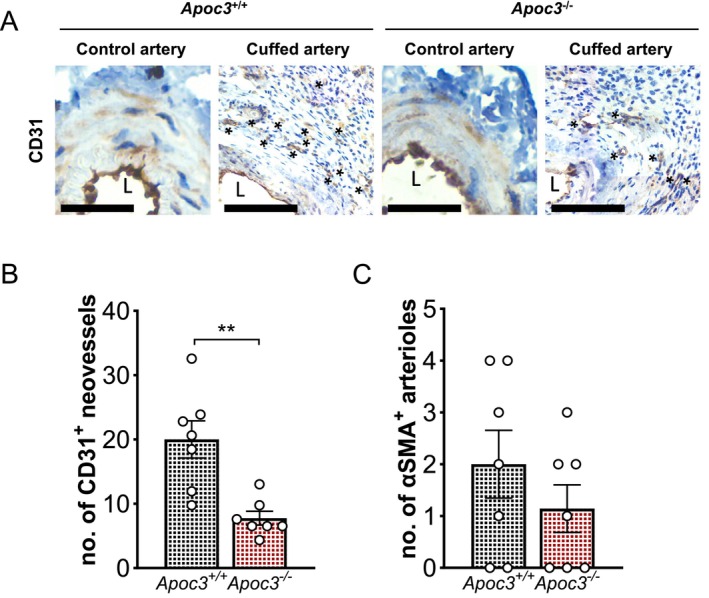
*Apoc3* deficiency inhibits neovascularisation in the peri‐arterial femoral cuff model of inflammatory angiogenesis. (A) Representative brightfield images of control and cuffed femoral arteries (black arrow) and surrounding adventitia stained with CD31 (brown; *) to detect neovessels in *Apoc3* ^+/+^ and *Apoc3* ^−/−^ mice. Scale bars = 50 μM. (B) Number of CD31^+^ neovessels detected in the adventitia of the cuffed femoral arteries of *Apoc3* ^+/+^ and *Apoc3* ^−/−^ mice. (C) Number of α‐Actin^+^ arterioles detected in the adventitia of the cuffed femoral arteries of *Apoc3* ^+/+^ and *Apoc3* ^−/−^ mice. Values are expressed as mean ± SEM (*n* = 7 per group). **p ≤ 0.01 for Student's *t*‐test.

After 21 days, cuff placement caused an expansion of adventitia around the femoral artery compared to control arteries (Figure 2A). We also observed the development of neovessels surrounding the cuffed femoral arteries of *Apoc3* ^+/+^ mice, as indicated by CD31^+^ staining; however, this response was blunted by ~40% in mice with global ApoC3 deletion (Figure 1B). Assessment of the number of adventitial α‐smooth muscle actin (SMA)^+^ arterioles in response to cuff placement around the femoral artery was measured to determine whether there was formation of established vessels. These were evident in 5/7 of the *Apoc3* ^+/+^ mice and 4/7 of the *Apoc3* ^−/−^ mice, and there was a trend for a reduction in *Apoc3* ^−/−^ mice (Figure 2C).

Altogether, these findings support a role for ApoC3 in promoting inflammation‐induced neovascularization.

### 3.3 ApoC3 Stimulation of THP‐1 Monocytes Induces Tubulogenesis and VCAM‐1 Expression in HUVECs

**FIGURE 3:**
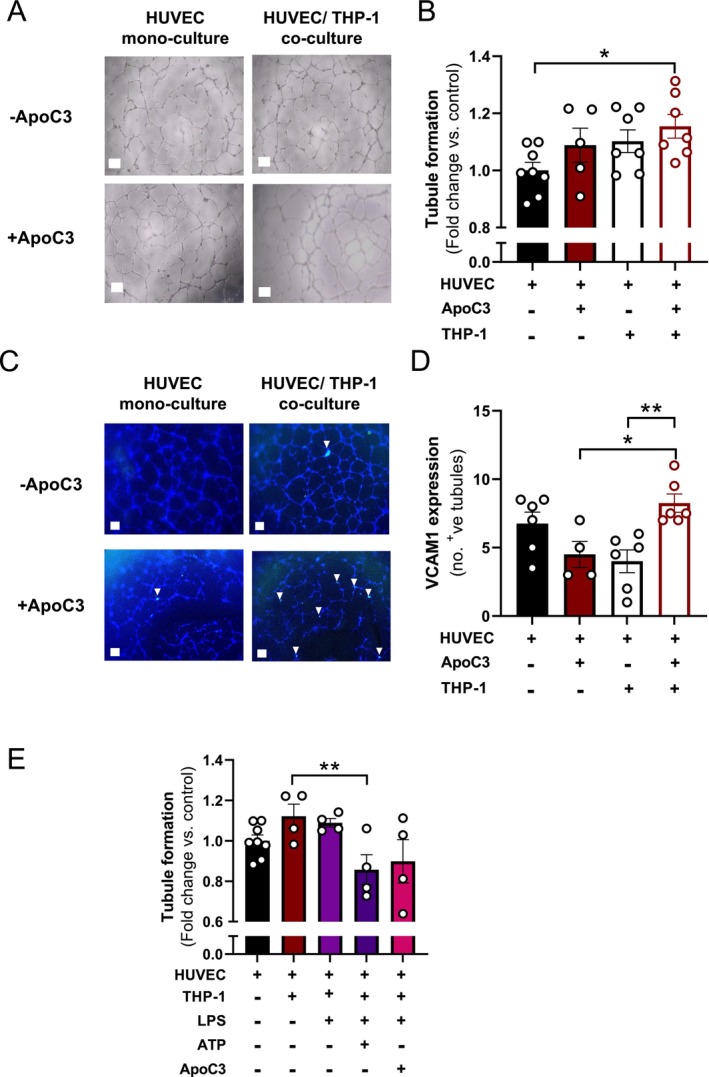
Human ApoC3 stimulation of THP‐1 monocytes is effective in inducing tubulogenesis and VCAM‐1 expression in HUVECs. (A) Representative images of intrinsic tubule formation in ECM of HUVECs and HUVECs co‐cultured with THP‐1 monocytes (2.5 × 10^4^ cells) with and without ApoC3 treatment (50 μg/mL). (B) Quantification of the number of tubules formed by HUVECs following ApoC3 treatment and/or THP‐1 monocyte co‐culture compared to control HUVECs. (C) Representative images of VCAM1 immunofluorescence staining (green, white arrow; DAPI in blue) of tubules formed by HUVECs in ECM in the presence and absence of ApoC3 and THP‐1 monocytes. (D) Quantification of the number of tubules expressing VCAM‐1 in control wells (HUVECs only) and following addition of ApoC3 and/or THP‐1 monocytes. (E) Tubule formation of HUVECs in control wells, or with THP‐1 monocytes treated with ApoC3, LPS (50 μg/mL), or ATP (5 mM). Scale bars = 50 μM. Values are expressed as mean ± SEM (*n* = 4–8 per group). **p < 0.01 and *p < 0.05 for one‐way ANOVA.

To investigate the role of ApoC3 in inflammation‐induced angiogenesis, we conducted co‐culture studies in which HUVECs seeded on ECM‐coated wells were allowed to undergo tubulogenesis in the presence or absence of THP‐1 cells and ApoC3. While both ApoC3 in monoculture and THP‐1 cells in co‐culture tended to increase tubule formation, it was only after HUVEC‐THP‐1 co‐culture was performed in the presence of additional ApoC3 that there was a significant increase in tubule formation (Figure 3B; *p* < 0.05). Furthermore, the number of VCAM1^+^ tubules was increased when co‐culturing HUVECs with THP‐1 monocytes pre‐treated with ApoC3 compared to treating HUVECs with ApoC3 or co‐culturing HUVECs with THP = 1 monocytes alone (Figure 3C,D; *p* < 0.05). Previously, it has been suggested that ApoC3 can activate the NLRP3 inflammasome [21]. We compared ApoC3 in the presence of THP‐1 cells treated with LPS, with the traditional NLRP3 inflammasome activator, LPS + ATP. Here we saw that NLRP3 inflammasome activation impaired angiogenesis, with lower tubule formation after LPS + ATP treatment. However, in the presence of LPS + ApoC3, the anti‐angiogenic effect was not seen (Figure 3E).

### 3.4 ApoC3 Global Deletion Has No Effect on Ischaemia‐Driven Neovascularisation In Vivo

**FIGURE 4:**
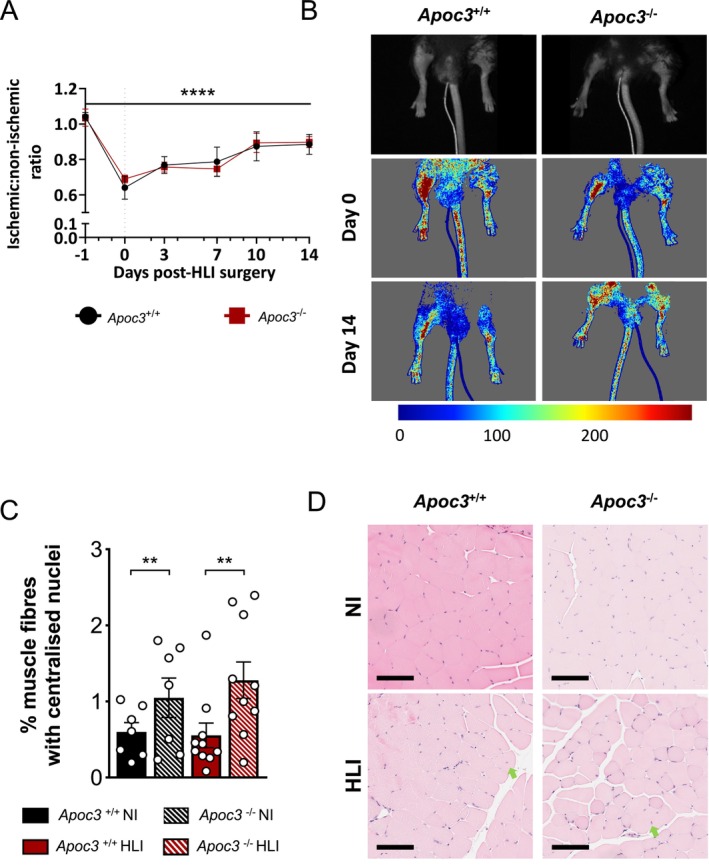
*Apoc3* deficiency has no effect on reperfusion or muscle fiber regeneration in ischaemia‐driven angiogenesis. (A) Calculated ratio of the Laser Doppler flux of ischemic (HLI) to non‐ischemic (NI) limbs following 14 days of hind limb ischemia in *Apoc3* ^+/+^ and *Apoc3* ^−/−^ mice and (B) representative images showing flux in hindlimbs from mice immediately post‐ligation and 14‐days post‐surgery. (C) Percentage of gastrocnemius muscle fibers with centralized nuclei 14 days post‐HLI surgery and (D) representative hematoxylin and eosin images showing fibers with centralized nuclei (green arrow). Scale bars = 100 μM. Values are expressed as mean ± SEM (*n* = 7–10 per group). ****p ≤ 0.0001 and **p ≤ 0.01 for two‐way ANOVA.

To understand the role of ApoC3 in ischaemia‐driven angiogenesis, we used the hind limb ischaemia model, whereby the left femoral vascular bed is ligated and excised, and the return of blood flow to the ischaemic limb is measured regularly over 14 days. Removal of the femoral vascular bed typically results in the increased expression of *Hif1a* and *Vegf1* in the gastrocnemius muscle in response to hypoxia [24]. Laser Doppler perfusion imaging revealed that prior to induction of ischemia, the blood perfusion ratio between the left and right limbs was comparable in both *Apoc3* ^+/+^ and *Apoc3* ^−/−^ mice (1.04 ± 0.02 vs. 1.04 ± 0.06; *p* > 0.05; Figure 4A,B).

**FIGURE 5:**
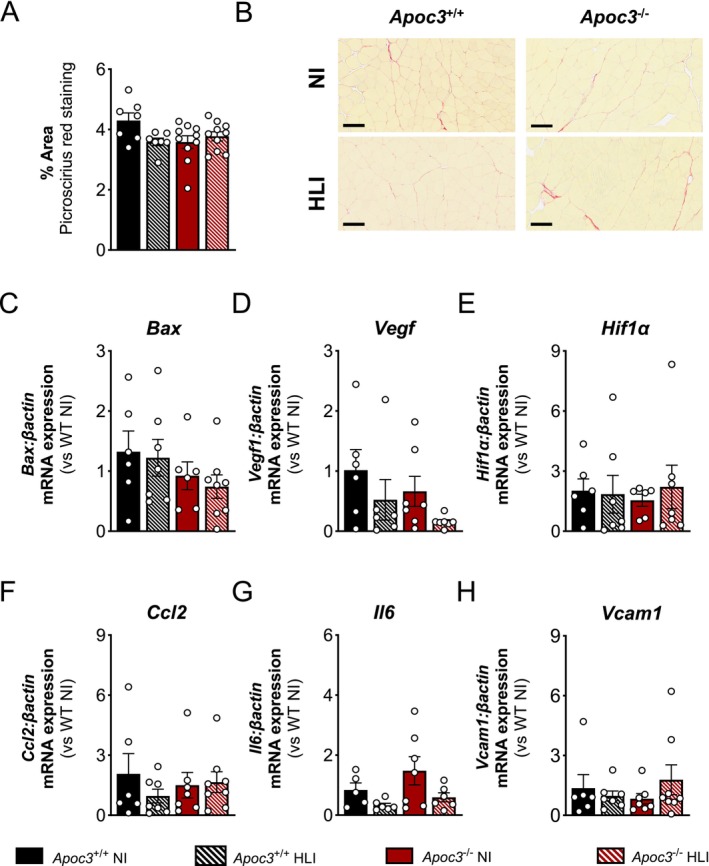
*Apoc3* deficiency has no effect on interstitial fibrosis, or the expression of angiogenic and pro‐inflammatory genes in the gastrocnemius muscle of ischemic limbs 14 days post‐HLI surgery. (A) Percentage area of collagen deposition and (B) representative brightfield microscopy images of gastrocnemius muscle sections stained with picrosirius red from ischemic (HLI) and non‐ischemic (NI) limbs of *Apoc3* ^+/+^ and *Apoc3* ^−/−^ mice. Scale bars = 100 μM. Gastrocnemius mRNA expression of (C) *Bax*, (D) *Vegf1*, (E) *Hif1a*, (F) *Ccl2*, (G) *Il6*, and (H) *Vcam1* from ischemic and non‐ischemic limbs of *Apoc3* ^+/+^ and *Apoc3* ^−/−^ mice 14 days post‐surgery. Values are expressed as mean ± SEM (*n* = 7–10 per group). *p* > 0.05 for two‐way ANOVA.

Following surgical ligation and removal of the femoral artery, blood flow was equally reduced in both *Apoc3* ^+/+^ and *Apoc3* ^−/−^ mice compared to the non‐ischaemic limb (0.64 ± 0.07 vs. 0.69 ± 0.03; *p* > 0.05; Figure 4A,B). There was no difference in the ischemic to non‐ischemic limb perfusion index between *Apoc3* ^+/+^ and *Apoc3* ^−/−^ mice at any point of the 21‐day treatment period. Consistent with these findings, there was evidence of muscle fiber regeneration in gastrocnemius muscles of both *Apoc3* ^+/+^ and *Apoc3* ^−/−^ mice in response to ischemia, as measured by the increased percentage of muscle fibers with centralized nuclei compared to the non‐ischemic limb (*p* < 0.01; Figure 4C,D). However, there were no differences in regenerating muscle fibers between *Apoc3* ^−/−^ and *Apoc3* ^+/+^ mice. Furthermore, there were no differences in fibrosis as measured by quantification of picrosirius red staining, mRNA expression of the apoptotic gene, *Bax*, the angiogenic genes, *Hif1a* and *Vegf1*, or proinflammatory genes *Ccl2*, *Il6*, or *Vcam1*, observed in response to ischaemia in the gastrocnemius muscles from either *Apoc3* ^+/+^ or *Apoc3* ^−/−^ mice (Figure 5A–H).

### 3.5 *Apoc3* Deficiency Has No Effect on Neovascularization

**FIGURE 6:**
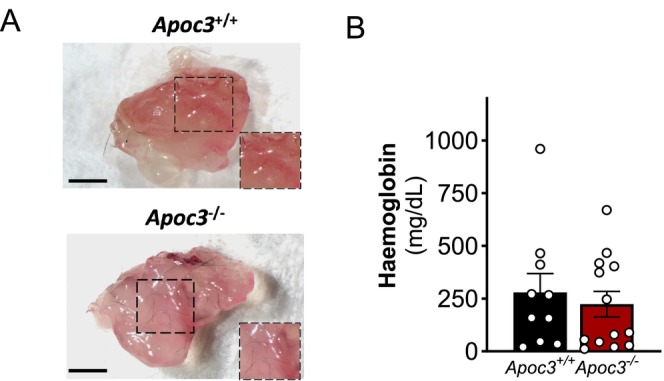
*Apoc3* deficiency has no effect on matrigel plug neovascularisation. (A) Representative images and (B) hemoglobin content of Matrigel plugs extracted from *Apoc3* ^+/+^ and *Apoc3* ^−/−^ mice 14‐days post‐injection. Scale bars = 50 μM. Values are expressed as mean ± SEM (*n* = 10–13 per group). *p* > 0.05 for Student's *t*‐test.

We investigated the influence of ApoC3 on neovascularisation using an in vivo assay whereby ECM plugs containing growth factor are subcutaneously injected above the femoral artery to promote neovessel growth. In this model, *Apoc3* ^−/−^ mice exhibited vascularisation of the ECM plug that was similar to *Apoc3* ^+/+^ mice (Figure 6A,B).

## 4 Discussion

In this study, we demonstrate that (i) *Apoc3* deficiency is associated with reductions in inflammation‐induced neovascularization expression of angiogenesis‐related genes (*Hif1α*/*Vegf1*) and inflammatory markers (*Rela*/*Cd68*), and (ii) preserved physiological, ischemia‐driven angiogenesis. These results identify ApoC3 as a critical regulator of inflammation‐induced angiogenesis, offering a potential novel role in vascular disease [29].

This study demonstrates that ApoC3 can induce inflammatory‐driven angiogenesis. A key strength of this study is that we showed a role for ApoC3 in inflammation‐induced angiogenesis at an acute timepoint (24 h post‐cuff placement), as evidenced by increased perivascular *Cd68* expression, accompanied by a marked increase in mRNA expression of angiogenic markers *Hif1a* and *Vegf1* in *Apoc3* ^+/+^ mice, which was absent in *Apoc3* ^−/−^ mice. This indicates that ApoC3 is responsible for driving the early macrophage recruitment to the injury site, which resulted in sustained angiogenesis 21 days post‐cuff placement in Apoc3^+/+^ mice, but not Apoc3^−/−^ mice. This pathway includes adhesion molecule activation on vascular endothelial cells, evidenced by our co‐culture studies with human cells, where ApoC3 stimulated greater endothelial cell tubule formation, which correlated with THP‐1 induced VCAM1 expression. These results align with previous reports implicating ApoC3 in macrophage recruitment by endothelial cells, whereby ApoC3 was shown to upregulate endothelial cell VCAM1 and Intercellular Adhesion Molecule 1 expression via a protein kinase‐C and NF‐κB‐dependent, lipid‐independent pathway [20]. Moreover, ApoC3 has been shown to increase NF‐κB‐directed expression of β1‐integrin in THP1 monocytes, promoting adhesion to endothelial cells [22]. ApoC3 has recently been shown to stimulate the NLRP3 inflammasome [21]; yet we saw no evidence of this, as tubule formation was reduced with NLRP3 activation using lipopolysaccharide (LPS) and adenosine triphosphate (ATP), but not in the presence of LPS and ApoC3. ApoC3 may participate in a vicious cycle in the context of inflammatory angiogenesis by promoting the recruitment of monocytes, which produce VEGF to promote excessive neovascularization [30, 31]. The newly formed vessels then facilitate an increased supply of macrophages to this site, further exacerbating inflammation and angiogenesis [32]. This effect is important in the context of atherogenesis, which in its primary stages, involves neovascularization in the adventitial vasorum [33]. While ischemic insults to the vessel wall may initiate plaque neovascularization, it is sustained by inflammatory‐driven angiogenesis and contributes to accelerated plaque development and instability [34]. The findings in this study demonstrate that *Apoc3* deficiency significantly reduces adventitial neovascularization in response to inflammation and highlights ApoC3 as an attractive target for improving plaque stability in the peripheral vasculature. ApoC3 antisense oligonucleotide treatment has been shown to be protective of progressive atherosclerosis in mice [35]. However, the current study is the first to demonstrate that *Apoc3* deficiency is protective in a model of pathological angiogenesis, and that this angiogenic effect is likely independent of decreased circulating triglycerides. In the context of atherosclerotic PAD, an increase in a glycosylated proteoform of ApoC3 was associated with a decreased ankle‐brachial index, which is diagnostic of PAD [29]. Little else is known about ApoC3 levels in PAD or whether it is correlated with disease severity, and this will be of interest to observe in future studies.

Despite demonstrating similarly lower triglyceride levels compared to *Apoc3* ^+/+^ mice in the hindlimb ischemia model, *Apoc3* deficiency did not affect ischemia‐driven angiogenesis, blood flow, or angiogenic marker expression in the hindlimb ischemia model of PAD. We posit ApoC3 is specifically involved in inflammation‐induced angiogenesis and not in response to ischemia. *Apoc3* deficiency resulted in lower *Cd68*, *NF‐κB*, *Hif‐1α*, and *Vegf1* expression under inflammatory conditions following femoral artery cuff implantation, suggesting ApoC3 influences angiogenesis only in the presence of macrophage infiltration. HIF‐1α and VEGF are key hypoxia‐induced angiogenic regulators. Under low oxygen, HIF‐1α avoids hydroxylation, translocates to the nucleus, and dimerizes with HIF‐1β to activate hypoxia response elements, inducing VEGF expression [36]. Tumor angiogenesis studies further revealed NF‐κB can drive VEGF expression via both HIF‐1α‐dependent and ‐independent pathways and engages in a negative feedback loop with HIF‐1α [37]. The complexity of this signaling pathway may explain the increased *Hif‐1α* and *Vegf1* mRNA expression in *Apoc3* ^+/+^ cuffed arteries 24 h post‐cuff placement. At 14 days post‐hindlimb ischemia surgery, angiogenic and inflammatory gene expression did not differ between ischemic and non‐ischemic limbs, likely due to the rapid recovery of *Apoc3* ^+/+^ and *Apoc3* ^−/−^ mice. Blood flow reached ~90% of baseline by day 10, reflecting the transient nature of angiogenic and inflammatory responses. Additionally, increased regenerating muscle fibers (fibers with centralized nuclei) in ischemic limbs compared to non‐ischemic limbs confirmed recovery from ischemia, with no difference between *Apoc3* ^+/+^ and *Apoc3* ^−/−^ mice. Furthermore, *Apoc3* deficiency did not enhance neovascularization in the presence of an ECM plug containing FGF‐2, which suggests that ApoC3 does not interfere with growth factor pathways associated with hypoxia‐induced angiogenesis [38]. Overall, these findings indicate that ApoC3 does not contribute to hypoxia‐driven angiogenesis.

Given the relationship between hypertriglyceridemia and CVD risk, it is not surprising that loss of function mutations in ApoC3 confer protection against the risk of ischemic vascular disease and atherosclerotic plaque development [39]. ApoC3 has therefore become an increasingly attractive target for reducing cardiovascular risk in patients with hypertriglyceridemia, with several antisense oligonucleotide therapies and hepatocyte‐targeted siRNAs being developed against ApoC3 [40, 41, 42, 43]. Each strategy has demonstrated significant triglyceride lowering effects (i.e., decreased by > 50% [41, 42]), and ongoing Phase 3 trials will further evaluate whether anti‐ApoC3 therapy reduces cardiovascular risk [44]. The reduction in circulating triglycerides on a normal diet in our mice is consistent with preclinical studies in *Apoc3* ^−/−^ rabbits. In these studies, plasma triglycerides were decreased by 50% compared to wild type, while lipoprotein lipase levels increased [45]. These preclinical results mirror observations in clinical studies, where loss of function mutations in the ApoC3 gene [13, 15], of which at least three variants have been identified, are associated with low levels of plasma triglycerides, and gain of function mutations in the ApoC3 gene are associated with a 32% increase in triglyceride levels compared to matched subjects [46]. While clinical development programs of agents that inhibit ApoC3 have exclusively focused on their ability to modulate atherogenic lipid parameters, they may exert additional effects at the level of the artery wall with the potential to contribute to reductions in cardiovascular risk.

Preclinical studies should also explore the role of triglycerides in angiogenesis to distinguish the triglyceride‐dependent and ‐independent effects of ApoC3 in inflammatory and hypoxic PAD models. In patients with low‐density lipoprotein (LDL)‐cholesterol lowering with statins but high residual CVD risk, plasma triglyceride levels greater than 150 mg/dL were associated with an increased need for peripheral revascularization surgery [47], confirming a role for triglycerides in PAD. Despite evidence implicating triglycerides in the development of atherosclerosis, therapies aimed at reducing triglyceride and VLDL synthesis, such as fibrates and omega‐3 fatty acids, have yielded mixed results in preventing CV events [48]. Recent trials, such as PROMINENT (fibrate) and STRENGTH (omega‐3 fatty acids) [49, 50], demonstrated the triglyceride‐lowering effects of these drug classes but were prematurely halted due to a failure to prevent major adverse cardiovascular events. While the REDUCE‐IT trial demonstrated a reduction in major cardiovascular events with icosapent ethyl (purified omega‐3 fatty acid, eicosapentanoic acid), this was not associated with triglyceride lowering, suggesting other factors that were likely contributing to this benefit [51]. Triglyceride‐rich lipoproteins (TRLs) contribute to atherosclerotic CVD by promoting atherogenic modifications in LDL and high‐density lipoproteins (HDL), inducing endothelial dysfunction through oxidative stress pathways and promoting the endothelial cell expression of pro‐inflammatory adhesion molecules and cytokines [52]. Thus, the combination of anti‐inflammatory and anti‐triglyceridemic activity with an ApoC3 inhibition strategy may prove superior to any prior therapy that has been trialed for PAD, and the effects of ApoC3 inhibition are likely to impact on coronary artery disease (CAD)‐related events in people with PAD and CAD as well.

Depending on the pathophysiological context, angiogenesis occurs via distinct, yet overlapping, pathways [53]. Pathological angiogenesis is driven by inflammatory stimuli, such as the transcription factor NF‐κB, which primarily promotes the recruitment of monocytes/macrophages [54] to the endothelium but is also implicated in the expression of cell proliferation genes [55]. VEGF is crucial to physiological angiogenesis in response to ischemia, as it is upregulated following HIF‐1α induced transcription, and its signaling contributes to endothelial cell proliferation, migration, and differentiation into tubular structures [6, 7, 8, 9, 56, 57]. VEGF is similarly upregulated in response to NF‐κB signaling [12]. Although VEGF‐A therapy was once thought to be an attractive treatment option in the context of PAD, it proved disappointing in clinical trials [10, 11], necessitating investigation into alternative mechanisms that contribute to angiogenesis in PAD. It would be of interest to determine whether ApoC3 inhibitor therapies reduce the incidence of PAD, particularly in individuals with chronic inflammation.

A key limitation of the study is that there is no true model to recapitulate PAD. Hindlimb ischemia is the most routinely used model for this purpose but is exhibits significant variation across mouse strains [58] and with modified surgical techniques [59]. In this study, we have a relatively mild model of hindlimb ischemia, with rapid reperfusion and no toe necrosis. Some have argued that this more accurately reflects the chronic ischemia in PAD [59], while a more severe insult is common [25]. It is also important to consider that the perfusion flux ratio to the contralateral limb is dependent upon experimental factors such as the level of anesthesia and body temperature, which affect both limbs. Our use of two models that capture different aspects of the disease process is a strength; however, the use of mice that were young and had relatively normal triglyceride levels is another limitation. Assessing ApoC3 inhibition in atherosclerotic and aged models of PAD could clarify its therapeutic potential for stabilizing plaques and limiting excessive neovascularisation.

In summary, this study demonstrated that ApoC3 plays a role in inflammation‐induced angiogenesis, whilst preserving ischemia‐driven physiological angiogenesis. These findings have implications for the development of therapies that inhibit ApoC3 in the context of atherosclerotic PAD. Further research into how ApoC3 modulates endothelial‐monocyte crosstalk may uncover new strategies for targeting inflammation‐driven vascular remodeling in PAD and other atherosclerotic vascular diseases.

## Author Contributions

J.M.T., P.B., and J.M. performed the investigation. J.M.T., P.B., J.M., B.A.D.B., and K.J.B. performed the formal analysis. C.A.B., B.A.D.B., S.J.N., and K.J.B. supervised the study. K.J.B. coordinated the project. J.M.T. wrote the original draft of the paper. All authors were involved in drafting and revising the manuscript. B.A.D.B. and K.J.B. should be considered joint senior authors.

## Conflicts of Interest

The authors declare no conflicts of interest.

## Data Availability

The data that support the findings of this study are available in the Sections 2 and 3.
